# Editorial: Metabolic regulation in cardiovascular homeostasis and disease

**DOI:** 10.3389/fcvm.2022.995207

**Published:** 2022-08-03

**Authors:** Kunhua Song, Zhong Wang, Kedryn K. Baskin

**Affiliations:** ^1^Division of Cardiology, Department of Medicine, University of Colorado Anschutz Medical Campus, Aurora, CO, United States; ^2^Gates Center for Regenerative Medicine and Stem Cell Biology, University of Colorado Anschutz Medical Campus, Aurora, CO, United States; ^3^Department of Cardiac Surgery, University of Michigan-Ann Arbor, Ann Arbor, MI, United States; ^4^Department of Physiology and Cell Biology, College of Medicine, the Ohio State University, Columbus, OH, United States

**Keywords:** metabolism, cardiac disease, biomarkers, metabolic remodeling, metabolic therapeutics

Metabolic dysregulation is a common feature in cardiovascular disease from congenital heart defects to heart failure. The Research Topic, metabolic regulation in cardiovascular homeostasis and disease, focuses on recent advances and challenges in understanding of metabolic regulation in cardiac physiology and pathogenesis. Here, a collection of 12 review and original research articles in this topic highlights recent findings and innovative approaches in the field of cardiovascular metabolism.

## Metabolic risks for cardiovascular disease

Many risk factors contribute to cardiovascular disease and mortality, such as obesity, aging, diabetes, metabolic syndrome, and obstructive sleep apnea (OSA). After following about one thousand participants, Liu et al. demonstrate that patients with metabolic syndrome and OSA have a higher risk of adverse cardiac events. These results may lead to the design of additional clinical studies to understand effects of multiple risk factors on cardiovascular disease. In a review article, Zhao and Liu summarize regulatory mechanisms of FOXO3, a critical regulator in aging-related vascular disease. Regarding roles of FOXO3 activation in vascular remodeling, FOXO3 has a potential for therapeutic targeting. Insulin resistance, a marker for metabolic syndrome also increases risk of heart disease. After investigating 240 non-diabetic patients with ST-segment elevation myocardial infarction (STEMI), Kasem et al. demonstrate that acute insulin resistance is associated with microvascular injury and poor hospital outcome in these patients. This study suggests that molecules related to insulin resistance may serve as predictors of microvasculature injury in myocardium of patients post-coronary angiography and angioplasty. Abnormal cholesterol levels are also a marker of metabolic syndrome. Increased level of cholesterol is a risk factor for coronary heart disease (CHD). Treatment with statin and its derivative drugs is used to lower cholesterol levels, indicated by fasting level of low-density lipoprotein cholesterol (LDL-C) <1.4 mmol/L and non-high-density lipoprotein cholesterol (non-HDL-C) level <2.2 mmol/L. Measuring non-fasting levels of LDL-C and non-HDL-C has been recommended to prevent ischemic events. Guo et al. have measured fasting and non-fasting levels of LDL-C and non-HDL-C in 397 patients with CHD. This study shows that much lower level of LDL-C in non-fasting phase shall be considered as a healthy threshold.

Many factors contribute to alterations of metabolism in the pathogenesis of cardiovascular disease. Ketema and Lopaschuk provide a comprehensive review of one specific-regulatory mechanism mediated by post-translational acetylation of non-histone proteins, such as mitochondrial proteins. This review article highlights the importance of unraveling interconnections among metabolic protein acetylation, fatty acid β-oxidation, and glucose oxidation in the heart under physiological and pathological conditions.

Quantification of metabolites and measurement of metabolic dynamics in the heart could facilitate identification of biomarkers for cardiovascular disease and understanding disease pathogenesis. Using gas and liquid chromatography-mass spectrometry, Aa et al. have made efforts to identify metabolites that predict risk of myocardial infarction (MI) by profiling plasma metabolites in 85 patients with MI chest pain, 61 patients with non-MI chest pain, and 84 control subjects. This study demonstrates that higher plasma levels of deoxyuridine, homoserine, and methionine increase the risk for MI. Verification of these findings in large samples would benefit prognosis and prevention of MI. Patients who suffer from congenital heart disease with single-ventricle (SV-CHD) are susceptible to heart failure (HF). Identification of biomarkers to predict HF risk in patients with SV-CHD would benefit timely care management. Xu et al. have measured oxygen consumption rates (OCR) in peripheral blood mononuclear cells (PBMCs) isolated from human subjects with SV-CHD (*n* = 20), biventricular CHD (BV-CHD, *n* = 16), and healthy control (*n* = 22). SV-CHD patients with HF show higher maximal respiratory capacity and respiratory reserve in PBMCs, while SV-CHD patients without HF show less maximal respiratory capacity. Because the authors collected PBMCs from relatively small sample sizes, more studies are needed to verify whether alterations of mitochondrial respiration in PBMCs could serve as a biomarker for HF risk in patients with SV-CHD. Powerful approaches and mathematical tools are critical to accurately measuring metabolic dynamics in heart tissue. Karlstaedt summarizes techniques and concepts for *in vivo* or *ex vivo* stable isotope labeling in measuring metabolic flux in cardiovascular research and advancements in analytical methods at the tissue and single-cell levels. Consideration of challenges in accurate measurement of metabolic flux in clinics studies would assist with timely management of disease, estimation of treatment efficacy, and decision on therapeutic strategies.

## Therapeutics targets of metabolic pathways

Dysregulation of metabolism is common in cardiovascular disease. Restoration of metabolic homeostasis has been regarded as a potential therapy for the disease. Obesity has been identified as a major risk factor of hospitalization, severe illness, and mortality of COVID-19 patients. Treatment of obesity plays an important role in ending the COVID-19 pandemic. Dickey et al. discuss metabolic impact of various treatments for obesity, including diet and calorie restriction and bariatric surgeries and highlight future studies needed to understand mechanisms of restoring metabolic homeostasis in obese patients by these treatments. Heart failure is accompanied by increased inflammation and glycolysis in myocardium. Wang et al. demonstrate that treatment with MCC950, a selective inhibitor of the inflammasome component NLRP3, ameliorated cardiac function and remodeling in obese mice with heart failure. This study indicates interactions between inflammation and metabolic alterations during cardiac pathogenesis in obese subjects. In another report, Chen et al. demonstrate that post-MI mice treated with 2-Deoxy-D-glucose (2-DG) show significantly decreased activation of cardiac fibroblasts and cardiac fibrosis. Although treatment with 2-DG 4 days post-MI significantly decreases cardiac fibrosis in mice, the treatment is not enough to ameliorate cardiac function and mortality. It is likely that cardiac fibrosis post-MI is activated by more than one pathway. Therefore, inhibition of only one pathway (e.g., glycolysis) appears insufficient to produce meaningful clinical outcomes. Reduction in ATP generation usually occurs in failing myocardium. Exogenous creatine phosphate (CrP), a high-energy phosphate provides cardiac protection by re-fueling ATP, attenuation of intracellular Ca^2+^ overload and oxidative stress, and anti-arrhythmias and platelet aggression in animal models. However, CrP does not improve long-term survival of patients with heart disease in various clinical trials. Yi-Dan et al. provide an overview of CrP physiology and pharmacological effects of exogenous CrP on ischemic myocardium. Yi-Dan et al. also summarize potential reasons why clinical benefits have not been observed in clinical trials, e.g., administration routs/dosage and lack of multicenter studies. Ultimately, treatment of cardiovascular disease by targeting metabolic dysregulation holds promising, but needs extensive and collaborative studies.

## Summary

Articles presented in this Research Topic provide in-depth insights into the relationship between metabolic homeostasis and cardiovascular pathogenesis. This editorial briefly summarizes each article in the collection. We are enthusiastic that this work will lead to the development of new approaches and discovery of novel mechanisms in the field of cardiovascular metabolism, which eventually results in designing of effective therapeutics for cardiovascular disease.

## Author contributions

KS, ZW, and KB contributed to the writing of the manuscript. All authors contributed to the article and approved the submitted version.

## Funding

This work was supported by the NIH R01HL133230, R01HL139735, R01HL163672, and K01DK116916.

## Conflict of interest

The authors declare that the research was conducted in the absence of any commercial or financial relationships that could be construed as a potential conflict of interest.

## Publisher's note

All claims expressed in this article are solely those of the authors and do not necessarily represent those of their affiliated organizations, or those of the publisher, the editors and the reviewers. Any product that may be evaluated in this article, or claim that may be made by its manufacturer, is not guaranteed or endorsed by the publisher.

